# Chronischer Schmerz als existenzielle Herausforderung

**DOI:** 10.1007/s00482-022-00632-2

**Published:** 2022-03-14

**Authors:** Kristin Kieselbach, Dominik Koesling, Thomas Wabel, Ursula Frede, Claudia Bozzaro

**Affiliations:** 1 Interdisziplinäres Schmerzzentrum ISZ, Albert-Ludwigs-Universität Freiburg im Breisgau, Breisacher Str. 117, 79106 Freiburg, Deutschland; 2 Institut für experimentelle Medizin, Arbeitsgruppe Medizinethik, Christian-Albrechts-Universität zu Kiel, Preusserstr. 1–9, Merkurhaus, 24105 Kiel, Deutschland; 3 Lehrstuhl für Systematische Theologie, Otto-Friedrich-Universität Bamberg, Bamberg, Deutschland; 4 Hofgasse 2a, 78337 Öhningen, Deutschland

**Keywords:** Behandlung chronischer Schmerzen, Existenzielles Widerfahrnis, Verzweiflung, Fragen der Sinngebung und Neuorientierung, Biopsychosoziales Schmerzmodell, Chronic pain treatment, Existential experience, Despair, Questions of meaning and reorientation, Biopsychosocial model of pain

## Abstract

Das biopsychosoziale Schmerzkonzept stellt gegenwärtig den Schwerpunkt schmerztherapeutischer Behandlungsprogramme dar. Jedoch kann damit die Komplexität chronischer Schmerzen, insbesondere ihre Bedeutung für die Betroffenen, nur unzureichend erfasst werden. Denn ein Kernaspekt des Phänomens *chronischer Schmerz *wird bislang nur in Einzelfällen berücksichtigt: sein *existenzieller Charakter*. Chronische Schmerzen können das Selbst- und Weltverständnis, die Lebenswünsche und -ziele, letztlich die gesamte Integrität der Betroffenen bedrohen. Selbstaussagen Erkrankter zeigen, dass chronischer Schmerz immer ein existenzielles Widerfahrnis darstellt und den Menschen in seiner Gesamtheit erfasst. Dies wird durch zwei Aspekte deutlich: zum einen durch die existenzielle *Verzweiflung am Schmerz*, zum anderen durch *Fragen der Sinngebung und Neuorientierung*. Allerdings berücksichtigen gängige Therapiekonzepte den existenziellen Charakter mit derartigen Herausforderungen bislang nicht adäquat. Chronischer Schmerz sollte daher stets unter einer umfassenden Perspektive wahrgenommen und behandelt werden. Hierbei sind die Aspekte *Einzigartigkeit anerkennen, zum Ausdruck verhelfen *und *dem Er-leben Raum geben *zur Unterstützung Erkrankter in ihrer Auseinandersetzung mit dem Schmerz besonders zu berücksichtigen.

## Abstract (en)

There is currently an emphasis on the biopsychosocial concept of pain in pain therapy programs. However, the complexity of chronic pain, in particular its importance for those affected by it, can only be insufficiently captured with this concept. This is due to the fact that, to date, one core aspect of the phenomenon *chronic pain *has only rarely been taken into account: its *existential character*. Chronic pain can threaten the self-image and the individual’s understanding of the world, their wishes and goals in life, and ultimately the entire integrity of those affected. Statements by chronic pain sufferers show that such pain always represents an existential experience and affects the person as a whole. Two aspects make this very clear: *the existential despair of the pain *on the one hand, as well as *questions of meaning and reorientation *on the other. Current treatment concepts, however, do not adequately consider the existential character of such challenges. Chronic pain should therefore always be perceived and treated from a holistic perspective. In this context, the aspects of *recognizing its uniqueness*, *helping to express the pain *and *giving space to the experience *are to be given special consideration in order to support chronic pain patients in dealing with their pain.

## Einführung

Sowohl in der Forschung als auch in der Behandlungspraxis ist bekannt, dass Schmerzen weitaus mehr sein können als ein rein physiologisches Problem, welches sich allein durch biomedizinische Ansätze therapieren ließe. Gerade chronische Schmerzen können eine komplexe biopsychosoziale Herausforderung darstellen. Auf dieser Erkenntnis aufbauend sind Behandlungsansätze wie die multimodale Schmerztherapie entwickelt worden. Dennoch wird ein Kernaspekt des Phänomens *chronischer Schmerz* bislang nur in Einzelfällen berücksichtigt: sein *existenzieller Charakter*. Chronische Schmerzen können das Selbst- und Weltverständnis, die Lebenswünsche und -ziele, letztlich die gesamte Integrität der Betroffenen bedrohen. Unter diesem Gesichtspunkt soll der chronische Schmerz im vorliegenden Text betrachtet werden.

Den Einstieg hierbei bildet eine Skizze der Grenzen multimodaler Schmerztherapie und des biopsychosozialen Schmerzmodells. Darauf aufbauend wird unter Bezugnahme auf das Total-Pain-Konzept dargelegt, weshalb ein holistisches Verständnis des chronischen Schmerzes notwendig ist. Ausgehend von Selbstaussagen Erkrankter wird sodann herausgearbeitet, inwiefern der chronische Schmerz immer ein existenzielles Widerfahrnis ist, das den Menschen in seiner Gesamtheit trifft und sein Leben aus den Fugen heben kann. Vor diesem Hintergrund wird in einem fünften Schritt umrissen, wie der existenzielle Charakter chronischer Schmerzen in der Schmerztherapie angemessen berücksichtigt werden kann, bevor abschließend ein kurzes Fazit gezogen wird.

## Die Grenzen der multimodalen Schmerztherapie und des biopsychosozialen Schmerzmodells

In Klinik, Forschung und gutachterlicher Praxis war es lange Zeit Usus, chronische Schmerzen physiologisch als eine hauptsächlich biomedizinisch verursachte und therapierbare Erkrankung in den Blick zu nehmen [27]. Diese Perspektive hat sich als vereinseitigend und reduktionistisch erwiesen. Heute gilt das biopsychosoziale Modell als das gängigste Konzept zur Erklärung chronischer Schmerzen und die interdisziplinäre multimodale Schmerztherapie als aktuell bestes Behandlungskonzept (z. B. [28]). Der gegenwärtige Deutungs- und Behandlungsansatz wird zudem dadurch verstärkt, dass der chronische, *beeinträchtigende* Schmerz [21] als eigenständige Krankheit begriffen wird. Diesem Verständnis tragen sowohl erweiterte Definitionen des chronischen Schmerzes [38] als auch die im Mai 2019 verabschiedete 11. Version der Internationalen Klassifikation der Krankheiten (ICD-11) Rechnung [47].

Allen Errungenschaften dieser Entwicklungen zum Trotz besteht die Notwendigkeit, die bisherige multidimensionale Perspektive hinsichtlich weiterer Aspekte zu überprüfen. Begründet wird dies nicht zuletzt durch die aktuelle Struktur und Durchführung der genannten multimodalen Schmerztherapie. Sowohl Diagnostik als auch Therapie orientieren sich an einem Paradigma, wonach chronische Schmerzsyndrome hauptsächlich auf biologische, psychische und soziale Faktoren zurückgeführt werden. Auf Behandlungsseite werden diese Dimensionen durch die jeweils beteiligten ärztlichen und therapeutischen Professionen und ihre spezifischen Ausrichtungen repräsentiert. Dies impliziert, dass der chronische Schmerz und seine biopsychosozialen Facetten seitens des Behandlungsteams im ersten Schritt in einer hauptsächlich fachbezogenen Sichtweise fokussiert werden. Daraus kann ein zunächst fragmentierter Eindruck des multifaktoriellen Schmerzsyndroms resultieren. Die Fusion der Einzelwahrnehmungen in Teambesprechungen und gemeinsamen Visiten zur interdisziplinären und interprofessionellen Teamleistung ist daher grundlegend, um die Schmerzerkrankung in ihrer ganzen Bedeutung zu erfassen. Angestrebt wird ein größtmögliches Verständnis des chronischen Schmerzes, seiner einzelnen Facetten und ihres Zusammenspiels – mit dem Ziel, dieses Zusammenspiel schließlich in den verschiedenen therapeutischen Maßnahmen adäquat und patientenindividuell zu berücksichtigen. Dieses bisherige Schmerzverständnis greift jedoch, wie auch in jüngeren Überlegungen zu adäquaten Schmerzmodellen deutlich wird, *perspektivisch* zu kurz (z. B. [6, 30]).

Die Weltgesundheitsorganisation (WHO) empfiehlt bereits seit 1984, das biopsychosoziale Modell um eine „spirituelle Dimension“ zu erweitern [36]. Diese Empfehlung wird heute durch zahlreiche Studien gestützt, die belegen, dass chronisch schmerzkranke Menschen auch existenzielle, spirituelle und religiöse Bedürfnisse haben (z. B. [2, 5, 40, 43]) und sie sich die Einbeziehung solcher Fragen in die Behandlung ausdrücklich wünschen [40, S. 334]. Dieser Wunsch steht im Gegensatz zur derzeitigen Realität schmerztherapeutischer Versorgung. Eine Untersuchung von Andersen et al. [4, S. 277] zur Arzt-Patienten-Kommunikation kommt zu dem Schluss, „that physicians rarely meet the existential, spiritual and religious needs of their chronic non-malignant pain patients“. Ungeachtet dieser Realität plädieren immer mehr Autoren für ein „biopsychosozial-spirituelles“ Modell, wie z. B. Gondo [18, S. 3], die in einer Untersuchung an 38 Schmerzpatienten zeigen konnte, „that greater existential well-being was associated with lower levels of pain severity and higher levels of global health“. In der Medizin gibt es bereits einen Bereich, der die Spiritualität explizit einbezieht – die Palliativmedizin. In Anlehnung an das von Cicely Saunders entwickelte Total-Pain-Konzept sollen Schmerzen nicht nur in ihrer biologischen, psychischen und sozialen, sondern auch in ihrer spirituellen Qualität wahrgenommen und behandelt werden [9, 23, 42, 49].

## Konturen einer erweiterten Perspektive auf chronischen Schmerz

Zentral für den ganzheitlichen Ansatz der Hospizpionierin Saunders ist die Beobachtung, dass „an individual’s pain is a whole overwhelming experience“ [50]. Diese Beobachtung ist nicht nur für die Palliativmedizin, sondern auch für die Therapie chronisch schmerzkranker Menschen von zentraler Bedeutung. Eine Betroffene beschreibt ihre Erfahrung mit folgenden Worten: „Früher konnte ich nur erahnen, wie verzweifelt jemand sein muss, der sich seines eigenen Lebens beraubt. Inzwischen erlebe ich diese Verzweiflung hautnah. Eine Verzweiflung, die mir die Luft nimmt, die mich lähmt, mich weinen, mich schweigen lässt“ [19, S. 73f]. Wie das Zitat zeigt, haben viele schmerzkranke Menschen nicht nur einen viel- oder mehrdimensionalen Schmerz – ihre Existenz wird in ihren Grundfesten erschüttert, ihr gesamtes Leben aus den Fugen gehoben. Davon ausgehend besteht unser Anliegen nicht in einer Auseinandersetzung mit einzelnen Aspekten chronischer Schmerzen, vielmehr möchten wir das Bewusstsein für ihren existenziellen Widerfahrnischarakter fördern.

Das Schmerzerleben ist etwas anderes als eine additive Zusammensetzung einzelner Komponenten. Allein schon deshalb, weil die Erfahrung existenzieller Erschütterung jede Facette des Lebens durchdringt und beeinflusst. Auf der Grundlage dieser wechselseitigen Verwobenheit plädieren wir dafür, die fragmentierte Sicht auf den Schmerz in eine ganzheitliche Erfassung und Beschreibung zu überführen. Der existenzielle Charakter chronischen Schmerzes wird daran deutlich, dass er *alle Lebensbereiche* der Erkrankten durchdringt und gleichsam einfärbt. Der Begriff des Existenziellen ist freilich ebenso komplex wie mehrdeutig, weshalb vorab einige definitorische Klärungen nötig sind. Sowohl zwischen Denkern der Existenzphilosophie (z. B. Martin Buber, Albert Camus, Martin Heidegger, Karl Jaspers, Søren Kierkegaard, Jean-Paul Sartre) als auch zwischen Vertretern existenzieller Psychotherapieansätze (z. B. Ludwig Binswanger, Viktor Frankl, Rollo May, Irvin Yalom) gibt es mehr oder minder deutliche Unterschiede. Ohne auf diese Differenzierungen eingehen zu können, verwenden wir den Begriff „existenziell“ hier im allgemeinen Sinne: Gemeint ist damit all das, was das individuelle Leben in seinen wesentlichen Aspekten (Zentralitätsaspekt) in einer allumfassenden Weise (Totalisierungsaspekt) betrifft.

Der so verstandene Begriff fokussiert menschliches Leben und Erleben in einer Perspektive, die im palliativmedizinischen Kontext auch durch Begriffe wie *spirituell* [23, 34] oder in Kombination der Begriffe als *existenziell und spirituell* mit thematisiert wird [4, 39, 43]. Sieht man von dem sehr spezifischen Phänomen des „spirituellen Schmerzes“ ab [35], so lassen sich die Begriffe *existenziell* und *spirituell* folgendermaßen aufeinander beziehen: Gemeinsam ist beiden Begriffen, dass sie auf die Gesamtheit menschlichen Erlebens zielen. Voneinander differenzieren lassen sie sich aus unserer Sicht durch die Unterscheidung von Erleben und dessen Bearbeitung: *Existenziell* bezieht sich auf das* Selbsterleben *des Menschen, das in seiner Zentralität nahezu alle Lebensbereiche durchdringt – negativ wie positiv. Dieses grundlegende Erleben wird – so unsere Annahme – von allen Menschen geteilt, auch wenn es nicht immer bewusst wahrgenommen oder thematisiert wird. *Spirituell* bezieht sich dagegen auf *eine* mögliche *Weise der Auseinandersetzung* mit diesem existenziellen Erleben. Ohne uns hier mit der komplexen Diskussionslage um die Abgrenzung zwischen Spiritualität und Religiosität auseinanderzusetzen [3, 29, 33, S. 504–509], gehen wir davon aus, dass sich die Bearbeitung eigenen Erlebens in Deutung und Sinnsuche durch Rückgriff auf überlieferte Religionen ebenso vollziehen kann wie in bewusster Abgrenzung von diesen, durch Ausübung von Elementen unterschiedlicher Traditionen oder Nutzung kultureller Angebote aus den Bereichen Literatur, Kunst oder Musik. So verstehen wir Spiritualität in sehr allgemeiner Weise als „Inbegriff derjenigen […] *Deutungsvollzüge, Praktiken bzw. Interaktionen, die sich für die Beteiligten auf das wesentliche Menschsein (in seinen uneinholbaren Ganzheitsmomenten) beziehen*“ [33, S. 517, Hervorhebung im Orig.].

Da wir im vorliegenden Text die Aufmerksamkeit vor allem auf den *existenziellen Charakter* des chronischen Schmerzes lenken möchten, sollen die mit ihm verbundenen Herausforderungen im Folgenden ausgehend von Selbstaussagen Betroffener herausgearbeitet werden.

## Der chronische Schmerz als existenzielle Herausforderung

Chronischer Schmerz führt zum Verlust von Alltagsgewohnheiten und Freizeitaktivitäten, geht oft mit dem Verlust des Arbeitsplatzes, aber auch von sozialen Kontakten einher, verändert die sozialen Rollen in der Familie oder im Freundeskreis. Selbstwert- und Identitätserleben geraten ins Wanken, weil Vorstellungen und Lebensziele, auf deren Verwirklichung die Betroffenen hingelebt haben, womöglich (so) nicht mehr erreichbar sind. Erfahrungen dieser Art gehören zu den existenziellen Herausforderungen chronischer Schmerzen. Mit diesem Begriff wird die Konfrontation mit Gegebenheiten unserer Existenz umschrieben, denen wir nicht entkommen können und für die wir keine Lösungen haben. Irvin D. Yalom [51] spricht in diesem Zusammenhang, angelehnt an Paul Tillich [46], von den „ultimate concerns of life“, zu denen insbesondere Tod, Isolation, Freiheit und Sinnlosigkeit gehören. Auch wenn chronisch schmerzkranke Menschen meist nicht von einem unmittelbar bevorstehenden Tod bedroht sind, so sind sie doch betroffen von Einsamkeit, Autonomieverlust und Sinnlosigkeit. Aus der Doppelperspektive einer Ärztin, die selbst zur betroffenen Patientin geworden ist, beschreibt Petrow [37, S. 190], wie sich die Konfrontation mit einer existenziellen Herausforderung anfühlen kann: „Mein inneres Koordinatensystem entsprach nicht mehr der aktuellen Situation. […] Woran […] konnte ich mich in mir selbst und in der Außenwelt (weiterhin) orientieren? Auf welche Eigenschaften konnte ich vertrauen, welche Fähigkeiten würden zurückkehren?“ Chronischer Schmerz ist keine vorübergehende Krankheit, die ohne Weiteres wieder in den eigenen Lebensverlauf integriert werden kann. Durch die anhaltende Omnipräsenz der Schmerzen sowie durch die Tatsache, dass sie in ihrer Intensität und in ihrem Aufkommen nicht immer gleichbleibend sind, wird der Umgang mit ihnen zu einer lebenslangen Aufgabe. Zu realisieren, dass ein Schmerzleiden chronisch ist und die gesamte weitere Lebenszeit (mit)bestimmen wird, ist ein radikaler Einschnitt in die eigene Lebensplanung. Plötzlich wird das Leben selbst fragmentiert wahrgenommen, geteilt in ein *Vorher* und ein *Nachher*, ähnlich der Situation nach einem schweren Unfall oder dem Verlust eines nahestehenden Menschen [37]. Gelebte Selbstverständlichkeiten werden erschüttert, die bislang zum kohärenten Selbstbild gehört haben: „[T]here’s an adaptation period […] which is extremely […] painful where it does feel as if your life is over. […] That seems like just running into a dead end entirely and you can’t see a way out“ (CP 31, w, 41 J., [22]). Der Weg aus dieser Sackgasse heraus führt nur über eine Neuorientierung: „Forget what you used to do. Forget how life used to be. Plan for a new and different, and equally rewarding life“ (CP 32, m, 57 J., [22]). Solche und ähnliche Selbstreflexionen, in denen sich Erkrankte deutend mit der eigenen Lebensgeschichte auseinandersetzen, stellen Versuche dar, angesichts der erlebten Brüche im eigenen Leben auf neue Weise Kohärenz zu stiften.

Geht man von Selbstaussagen Betroffener in Interviews [22, 31] und Autobiographien (z. B. [12, 19]) aus, so zeigt sich der existenzielle Charakter chronischer Schmerzen v. a. an zwei Aspekten: zum einen an der *Verzweiflung am Schmerz,* zum anderen an *Fragen der Sinngebung und Neuorientierung*. Exemplarisch für den Aspekt der Verzweiflung sind die Worte der Malerin Frida Kahlo [26, S. 69]: „… weil mir zu Hause niemand glaubt, dass ich wirklich krank bin. Ich darf nicht einmal davon sprechen, […] . Und so bin ich ganz alleine in meinem Leid und meiner Verzweiflung.“ Den Aspekt der Neuorientierung beschreibt eine Patientin so: „And I think that gradually over time it’s taken […] a couple of years for me to kind of properly get my life back and I feel like now that I have got my life back and although it’s not how I’d like it to be and I feel like I’m suffering […,] I am living the life that […] I almost would have led before“ (CP 16, w, 25 J., [22]).

In Theorie und Praxis der Schmerztherapie wird insbesondere der erstgenannte Aspekt der *Verzweiflung am Schmerz* selten erwähnt. Für das Leiden von Menschen angesichts der Erkenntnis, dass ein Teil ihres Ichs gebrochen oder gestorben ist, gibt es im Rahmen multimodaler Schmerztherapie kaum Raum. Der Schwerpunkt liegt auf einer möglichst frühzeitigen „Analyse und Modifikation dysfunktionaler Kognitionen“ sowie auf der „Anpassung körperlicher und sozialer Aktivitäten“ [13, S. 358, 356]. Der Blick der Behandelnden ist nach vorn auf die Zukunft gerichtet. Der Blick der Erkrankten dagegen verweilt noch in der Vergangenheit – bei dem, was sie verloren haben. Da die Chronifizierung von Schmerz schmerzpsychologisch v. a. mit dem Vorliegen dysfunktionaler Überzeugungen und Verhaltensweisen erklärt wird [20], besteht die Gefahr, dass Äußerungen der Verzweiflung und Trauer mitunter vorschnell pathologisiert, d. h. als Ausdruck unzureichender Krankheitsverarbeitung, als Anpassungsstörung, beschrieben werden. Eine Störung meint man, therapieren zu können. Dem existenziellen Leiden schmerzkranker Menschen aber weiß man zunächst nichts entgegenzusetzen – eine Erfahrung, die nicht zuletzt auch viele Therapeutinnen und Therapeuten als belastend erleben. Im Folgenden wollen wir deshalb kurz umreißen, wie Betroffene dabei unterstützt werden können, den Herausforderungen durch ihren Schmerz zu begegnen.

## Einbeziehung des existenziellen Charakters chronischer Schmerzen in die Schmerztherapie

Die Bedeutung des existenziellen Charakters chronischer Schmerzen ist bereits verschiedentlich untersucht worden (z. B. [4, 39, 45]). Aufgrund seiner Untersuchung an Palliativpatienten fordert Gabl [14, S. 29] für die Ausbildung professioneller Betreuungspersonen eine verstärkte Sensibilisierung „für das Thema Leiden und existenzielle Verzweiflung“. Doch abgesehen von den Arbeiten von Gebler [17] sowie von Gebler und Maercker [16] besteht weiterhin das Desiderat, den existenziellen Charakter chronischer Schmerzen auch in der Therapie von Menschen zu berücksichtigen, deren Sterben nicht unmittelbar bevorsteht. Geblers [17, S. 2] Studie zufolge führt eine solche Berücksichtigung „zu einem bedeutsamen therapeutischen Zugewinn“. Dieses Ergebnis unterstützt unser Plädoyer für eine Integration des existenziellen Charakters chronischer Schmerzen auch (und gerade!) bei ihrer Behandlung. Wobei wir uns nicht für eine *Abkehr* vom biopsychosozialen Behandlungsmodell einsetzen, vielmehr dafür, den chronischen Schmerz stets unter einer *umfassenden Perspektive* wahrzunehmen und zu behandeln. Folgende Aspekte sind dabei besonders hervorzuheben:

### Einzigartigkeit anerkennen

Zunächst ist auf einen scheinbar trivialen Aspekt hinzuweisen: den individuellen, nicht verallgemeinerbaren Charakter der Schmerzerfahrung, weshalb sich ihre Behandlung allgemeingültigen Konzepten entzieht. Beispielsweise kann das ganzheitliche Erfassen chronischer Schmerzen zur Folge haben, dass zwei Patienten mit ähnlichen Beschwerden durchaus unterschiedlicher Behandlungen bedürfen – weil ihre Lebenssituation unterschiedlich ist und ihrem Schmerz eine jeweils andere existenzielle Bedeutung zukommt. Wer mit chronisch Kranken arbeitet, ist wiederholt Situationen ausgesetzt, für die es keine vorgefertigten Lösungen gibt, die es zunächst einmal auszuhalten und mitfühlend zu begleiten gilt. Diese Begleitung ist nicht im Sinne reiner Passivität zu verstehen, vielmehr im Sinne des englischen Begriffs *„care“ *(= Betreuung, Pflege, Fürsorge)*, *definiert als aktives Bemühen um eine verbesserte Lebensqualität von Menschen, deren Leiderfahrungen sich großenteils nicht mehr ändern, bestenfalls lindern lassen. Die Behandlung von Schmerzen kann zur Überforderung für therapeutisches Personal und Erkrankte werden, wenn diese von der Vorstellung ausgehen, Schmerz sei prinzipiell kontrollierbar, man müsse eben nur die richtigen Mittel finden. Für die Unterstützung Betroffener bei ihrer Auseinandersetzung mit den existenziellen Herausforderungen ihrer Lage ist über bestimmte erlernbare Techniken (vgl. [24]) hinaus v. a. eine bestimmte innere *Haltung* entscheidend: die Bereitschaft zur persönlichen Reflexion existenzieller Themen sowie das Bemühen darum, sich auf das Leid der Erkrankten einzulassen und ihnen dabei zu helfen, ihren *eigenen* Umgang damit zu finden.

### Zum Ausdruck verhelfen

Existenzielle Leiderfahrungen betreffen den innersten Kern eines Menschen. Nicht alle Betroffenen dürften die Bereitschaft mitbringen, darüber im therapeutischen Kontext zu sprechen. Manche fürchten „eine psychiatrische Diagnose oder belehrende Ratschläge“ [37, S. 265]. Hier gilt es, Grenzen zu respektieren und die Angst vor Psychologisierung und Pathologisierung zu nehmen. Es gibt aber auch Schmerzkranke, denen die Ausdrucksmittel fehlen, um ihre existenzielle Betroffenheit zur Sprache bringen zu können. Für die Verarbeitung chronischer Schmerzen gibt es keinen Regelkatalog. Manchmal können die Berichte anderer Betroffener (wie z. B. in Patientenselbsthilfegruppen) Orientierungshilfe sein und der inneren Einsamkeit der Erkrankten entgegenwirken: „Endlich bin ich nicht mehr allein, es gibt noch andere, denen es genauso geht wie mir“ [10]. Auch kulturelle Ausdrucks- und Deutungsformen – etwa in Form von Literatur, Kunst und Musik, aber auch aus der Philosophie, der Religion oder aus dem, was (in bewusster Abgrenzung von überlieferten Religionen) als Spiritualität bezeichnet wird – können Patientinnen und Patienten dazu verhelfen, ihr existenzielles Erleben zum Ausdruck zu bringen [8, 26, 41].

Wie das gelingen kann, lässt sich am Beispiel der Klagepsalmen der jüdisch-christlichen Tradition veranschaulichen. Sie spiegeln eine ganze Bandbreite menschlicher Leidsituationen: Krankheit, Verfolgung, Vereinsamung, Verlust und Bedrohung. Das Leid wird weder erklärt noch bewertet. Alles Aufbegehren und alle Verzweiflung werden vor Gott gebracht: „Heile mich, ich bin am Ende meiner Kraft!“ (Ps 6,4). „Ich bin gefangen und weiß keinen Ausweg mehr“ (Ps 88,9). Die Worte „Mein Gott, mein Gott, warum hast du mich verlassen?“ (Ps 22,2) sind die letzten Worte Jesu am Kreuz (Markus 15,34). Mit seiner eigenen Klage etabliert Jesus das Recht des Klagens. Insbesondere für Menschen, denen es schwerfällt, ihre Verzweiflung in Worte zu fassen oder sich ihr eigenes Leid zuzugestehen, kann es in hohem Maße entlastend sein, wenn ihr Gegenüber ihnen einen Klagepsalm vorlegt. Unabhängig von der je eigenen Interpretation der Bibelstellen, unabhängig sogar davon, ob überhaupt eine religiöse Beheimatung vorliegt, wird an diesen Texten eines deutlich: Der Mensch darf seine Gefühle aussprechen, sein Leiden benennen – unzensiert. Wer in Klage und Anklage Widerstand zum Ausdruck zu bringen vermag, kann darin Widerhall erfahren und in Distanz zum Erlebten treten. Dies birgt die Chance, dass hieraus innere Ruhe, Trost und ein Impuls zur Neuorientierung erwachsen [11, 15] – ohne dass dieses Ziel damit automatisch erreicht werden muss [44, S. 111, 159].

Solche Artikulation ermöglicht, sich zum Erlebten ins Verhältnis zu setzen, indem die eigene Erfahrung eine Sprache findet [25, 48]. So lässt sich Betroffenen, die aufgrund ihrer Schmerzkrankheit zu verzweifeln drohen, ein Hilfsangebot an die Hand geben, ohne in Form eines therapeutischen Aktionismus vorschnell in den Modus der Optimierung ihrer Bewältigungsfertigkeiten überzugehen.

### Dem Er-leben Raum geben

„Nicht sich umzuorientieren, sondern die veränderte Situation zunächst wirklich zu erfassen, sie auszuhalten und um das Verlorene zu trauern, wäre für mich wichtig gewesen“ [37, S. 122]. Diese Äußerung einer Betroffenen weist darauf hin, dass Trauer nicht einfach übersprungen oder anderweitig kompensiert werden kann: „Gegen die Trauer hilft nur eins – trauern“ [1, S. 90]. Seitens der Helfenden steht vor allem eines im Vordergrund: dem chronischen Schmerz *standzuhalten, dazubleiben* und *anzuhören*, was auch immer an Trauer, Angst und Verzweiflung aus den Erkrankten herauskommen will. Wenn ein erkrankter Mensch sich mit belastenden Gefühlen auseinandersetzt, dabei jedoch wahrnehmen kann, „dass der Therapeut ihm weiterhin positiv zugewandt ist, ihn versteht und trotzdem ruhig bleibt“, kann davon ausgegangen werden, dass diese Haltung bei ihm „eine organismische Resonanz in Richtung auf mehr Ruhe erzeugt“ [32, S. 266]. Wer den Verlust des alten, schmerzfreien Lebens artikulieren, wer ihn betrauern kann, kann womöglich genau durch diesen Prozess einen ersten Schritt in Richtung auf ein erfülltes Leben gehen, in dem auch der Schmerz seinen Platz hat [7]. Dabei ist zu unterstreichen, dass der hier angedeutete Prozess kein linearer und einmaliger Vorgang ist. So, wie der Schmerz sich immer wieder in seiner Intensität und Bedrohlichkeit verändert, so wird auch die existenzielle Auseinandersetzung mit ihm immer wieder in einem eher zyklischen Prozess erfolgen. Trauer, Wut, Verzweiflung werden wiederholt auftreten, und es bleibt eine Lebensaufgabe, diese zu bewältigen.

### Multimodale Schmerztherapie weiterdenken

Chronischer Schmerz erfasst den betroffenen Menschen in Gänze – nicht nur als biopsychosozial bedingte Erkrankung, sondern als ein existenzielles Widerfahrnis. Die interprofessionelle und teamintegrierte Vorgehensweise in der multimodalen Schmerztherapie bietet fachspezifische Möglichkeiten, die patientenseitig sehr unterschiedlichen Bedürfnisse individuell wahrzunehmen und therapeutisch zu berücksichtigen. Die vielfältigen schmerzbeeinflussenden Faktoren unterliegen auf Patientenseite allerdings einem dynamischen Prozess, der auf Behandlungsseite auch als solcher erfasst werden muss. Es ist also erforderlich, im zeitlichen Verlauf sich verändernde Priorisierungen und deren Einflüsse auf das Schmerzerleben in das therapeutische Setting einzubeziehen und in einem jeweils neuen Gleichgewicht auszurichten. Wenngleich die multimodale Therapie äußeren Vorgaben und teils statischen Konzepten unterliegt, sollte sie ihr großes interprofessionelles Potenzial gerade in diesem Aspekt patientenindividuell nutzen. Die gesamte Belastung der Betroffenen, die durchaus veränderlich sein kann, sollte bedingungslos anerkannt werden und das therapeutische Vorgehen sollte daran situativ angepasst werden. Immer ist also eine Doppelperspektive auf das chronische Schmerzgeschehen einzunehmen, in der die bestmögliche therapeutische Entsprechung zu den veränderlichen ganzheitlichen Bedürfnissen auf Patientenseite grundlegend für eine gelingende gemeinsame Behandlungsperspektive ist.

## Fazit

Das biopsychosoziale Schmerzkonzept reicht nicht aus, um die Komplexität chronischer Schmerzen, insbesondere ihre Bedeutung für die Betroffenen erfassen zu können. Wir plädieren deshalb für eine explizite Berücksichtigung des existenziellen Charakters chronischer Schmerzen in deren Behandlung. Die Bedeutung des Schmerzes als ein Widerfahrnis zeigt sich v. a. an der existenziellen Verzweiflung am Schmerz sowie an Fragen der Sinngebung und Neuorientierung. Der Schwerpunkt derzeitiger Therapieprogramme liegt auf der Modifikation und Optimierung von Schmerzbewältigungsfertigkeiten der Erkrankten. Angesichts institutioneller Vorgaben und Erwartungen an objektiv darstellbare und möglichst schnelle Behandlungserfolge mag es schwerfallen, auch der Verzweiflung Zeit und Raum zu geben. Langfristig gesehen ist die Unterstützung des betroffenen Menschen in seiner Verzweiflung jedoch kein Zeitverlust, da die Auseinandersetzung damit zentrale Voraussetzung für seine weiteren Schritte ist, nicht zuletzt auch für den Schritt einer Neuausrichtung seines Lebens.
